# The association between sleep quality and accelerated epigenetic aging with metabolic syndrome in Korean adults

**DOI:** 10.1186/s13148-024-01706-x

**Published:** 2024-07-16

**Authors:** Ho-Sun Lee, Boram Kim, Taesung Park

**Affiliations:** 1 Forensic Toxicology Division, Daegu Institute, National Forensic Service, Andong-si, Gyeongsangbuk-do 39872 Korea; 2 Interdisciplinary Program in Bioinformatics, Seoul National University, Seoul, 08826 Korea; 3 Department of Statistics, Seoul National University, Seoul, 08826 Korea

**Keywords:** PSQI, Poor sleepers, Epigenetic age acceleration, DunedinPACE, Metabolic syndrome

## Abstract

**Background:**

Healthy sleep is vital for maintaining optimal mental and physical health. Accumulating evidence suggests that sleep loss and disturbances play a significant role in the biological aging process, early onset of disease, and reduced lifespan. While numerous studies have explored the association between biological aging and its drivers, only a few studies have examined its relationship with sleep quality. In this study, we investigated the associations between sleep quality and epigenetic age acceleration using whole blood samples from a cohort of 692 Korean adults. Sleep quality of each participant was assessed using the validated Pittsburgh Sleep Quality Index (PSQI), which encompassed seven domains: subjective sleep quality, sleep latency, sleep duration, habitual sleep efficiency, sleep disturbance, use of sleep medication, and daytime dysfunction. Four epigenetic age accelerations (HorvathAgeAccel, HannumAgeAccel, PhenoAgeAccel, and GrimAgeAccel) and the pace of aging, DunedinPACE, were investigated for epigenetic aging estimates.

**Results:**

Among the 692 participants (good sleepers [*n* = 441, 63.7%]; poor sleepers [*n* = 251, 36.3%]), DunedinPACE was positively correlated with PSQI scores in poor sleepers ($\gamma$=0.18, *p* < 0.01). GrimAgeAccel ($\beta$=0.18, *p* = 0.02) and DunedinPACE ($\beta$=0.01, *p* < 0.01) showed a statistically significant association with PSQI scores only in poor sleepers by multiple linear regression. In addition, every one-point increase in PSQI was associated with a 15% increase in the risk of metabolic syndrome (MetS) among poor sleepers (OR = 1.15, 95% CI = 1.03–1.29, *p* = 0.011). In MetS components, a positive correlation was observed between PSQI score and fasting glucose ($\gamma$= 0.19, *p* < 0.01).

**Conclusions:**

This study suggests that worsening sleep quality, especially in poor sleepers, is associated with accelerated epigenetic aging for GrimAgeAccel and DundinePACE with risk of metabolic syndrome. This finding could potentially serve as a promising strategy for preventing age-related diseases in the future.

**Supplementary Information:**

The online version contains supplementary material available at 10.1186/s13148-024-01706-x.

## Background

Over the last few years, the impact of sleep on overall health has garnered significant public attention, as obtaining healthy sleep is recognized as essential to optimal physical and mental well-being [1]. Sleep quality, including sleep latency and efficiency, plays a crucial role in numerous biological processes, such as circadian rhythms, hormone secretion, glucose homeostasis, and chronic diseases [2, 3]. For example, circadian rhythms are controlled by a biological clock located in the brain, and prolonged disruptions to the clock are associated with negative health consequences [4]. The associations between insufficient sleep duration and various health concerns, including obesity, elevated blood pressure, and an elevated risk of cardiovascular diseases, are well established [5, 6]. The accumulating evidence suggests that sleep loss and sleep disturbances play an important role as contributors to early disease onset and survival [7, 8].

As human life expectancy increases and the elderly population grows, there is a current trend of heightened attention toward age-related health conditions. One such condition is changes in sleep patterns across the lifespan, with older adults typically experiencing with a higher prevalence of sleep impairments in general population [9]. A growing body of research indicates that older individuals often experience more difficulty falling asleep and staying asleep compared to younger adults, with up to 50% of older people reporting difficulties in initiating and/or maintaining sleep [9]. Sleep disruption and insufficiency frequently occur among the elderly population and have been linked with dementia and all-cause mortality [10]. Although the precise mechanisms remain incompletely elucidated, the relationship between sleep patterns and the aging process may share comparable biological processes [11, 12]. Numerous studies have unraveled aging-related epigenetic modifications, including RNA modification, chromatin remodeling, and histone and DNA methylation [13]. Sleep deprivation may serve as both a sign of ill health and a trigger for epigenetic changes associated with biological aging [11]. Poor sleep quality, short sleep duration, and diagnosed insomnia have also been linked to shorter leukocyte telomere length, along with biological aging pathways such as increasing inflammation, DNA damage, and cellular senescence in varied populations of mid- to late-life adults [14]. Physiological stress and sleep deprivation-induced chronic inflammation have been associated with accelerating biological aging through epigenetic regulation [15–17]. However, the mechanism underlying their relationship remains inconclusive.

While a substantial body of research exists on the relationship between aging and sleep, investigations into the association of methylation age with and sleep quality are limited. In this study, we considered four epigenetic age (EA) markers: PhenoAge [18], GrimAge [19], HorvathAge [20], and HannumAge [21], and pace of aging marker, as measured by DunedinPACE [22], as candidate markers for sleep quality. We assessed self-reported sleep quality and investigated association between sleep quality scores and epigenetic age accelerations (EAAs) to identify potential causal relationship for chronic diseases in a Korean population.

## Material and methods

### Study participants

This cohort study, as a part of the KoGES (Korean Genome and Epidemiology Study), was conducted primarily to evaluate the association between lifestyle factors and genetic risk factors with the incidence of chronic diseases in a Korean population, which began in 2001 with 8,842 participants aged 40–69 years and was conducted every two years thereafter [23]. Our data were collected from 2009 to 2010, during which 701 participants were asked to complete questionnaires covering demographic information, lifestyle, medical history, and health conditions. All participants were residents of Ansan City in South Korea. The study was approved by the Institutional Review Boards of the National Institutes of Health of Korea and Seoul National University (IRB No. E2209/001-001).

### Data collection

To assess participants’ characteristics, a self-reported or interview-based questionnaire was administered, which included participant’s age, sex, smoking habit, alcohol consumption, physical activity, household income and medical history. Smoking status was categorized into current, former, and never smokers. Former smokers were defined as individuals who had ceased smoking and had a history of having smoked less than 400 cigarettes during their lifetime. Alcohol consumption was also categorized into current, former, and never drinkers. Former drinkers were defined as individuals who had abstained from consuming alcohol for a period of at least one year. Monthly household income, as an indicator of economic status, was classified into two groups: < 2 million South Korean Won (KRW) (approximately < 890 US dollar in 2018), and ≥ 2 million KRW. Physical activity was evaluated using metabolic equivalent of task (METs-hour/day) using the International Physical Activity Questionnaire [24]. Medical records pertaining to diagnosed conditions such as type 2 diabetes, hypertension, and dyslipidemia were recorded.

Biochemical data and anthropometric measurements, including blood pressure (BP), height, weight, and waist circumference (WC), were obtained using established methods [23]. Body mass index (BMI) was defined as body weight divided by the square of height in meters (kg/m^2^). Participants with missing data on at least one phenotype or methylation were excluded from the analysis.

### Metabolic syndrome definition

Metabolic syndrome (MetS) was defined according to the modified criteria of the National Cholesterol Education Program-Adult Treatment Panel III (NCEP-ATP III) with the appropriate WC cutoff points for central obesity in Korean population [25]. Diagnosis of MetS was defined if individuals exhibited at least three of the following components: (1) WC ≥ 90 cm for men and ≥ 85 cm for women, (2) triglyceride (TG) level ≥ 150 mg/dL or undergoing pharmacologic treatment, (3) high-density lipoprotein (HDL) cholesterol level < 40 mg/dL in men and < 50 mg/dL in women or undergoing pharmacologic treatment, (4) systolic/diastolic BP (SBP/DBP) ≥ 130/85 mmHg or receiving antihypertensive medication, and (5) fasting glucose (FAG) level ≥ 100 mg/dL or undergoing pharmacologic treatment.

### Assessment of sleep quality

Assessment of sleep quality was conducted using the Pittsburgh Sleep Quality Index (PSQI) self-rated questionnaires, which provide measures of 7 domains: (1) subjective sleep quality, (2) sleep latency, (3) sleep duration, (4) sleep disturbance, (5) sleep efficiency, (6) use of sleep medication, and (7) daytime dysfunction scores [26]. These domains are rated on a 3-point ascending scale, with 0-point indicating ideal sleep quality and 3-point indicating poor sleep quality. The global PSQI score, which ranges from 0 (indicating the best sleep quality) to 21 points (indicating the worst sleep quality), was calculated.

The PSQI assesses usual sleep habits (sleep quality and disturbances) over a 1-month time. For example, the PSQI survey evaluates habitual prolonged sleep latency rather than occasional instances of prolonged sleep latency. Based on the time taken to fall asleep each night, the questionnaire assigns a score ranging from 0 to 3 (0 = falls asleep in ≤ 15 min, 1 = falls asleep in 16–30 min, 2 = falls asleep in 31–60 min, and 3 = falls asleep in > 60 min). Sleep duration was obtained from a single-item question asking about typical sleep duration on the PSQI and fewer than 7 h per night was categorized as unhealthy sleep duration [27]. The global PSQI score > 5 indicated poor sleep quality, in accordance with published recommendations [26].

### Epigenetic age estimates

Blood-based DNA methylation (DNAm) levels at CpG sites were quantified using the Illumina HumanMethylationEPIC BeadArray (Illumina, Inc., San Diego, CA, USA), which covers over 850,000 CpG sites for 701 individuals. The DNAm data were preprocessed using R package ChAMP [28] based on the following exclusion criteria: (1) poor-quality samples with detection *p*-value less 0.01, (2) probes with fewer than three beads in at least 5% of samples per probe, (3) all SNP-related probes. Additionally, non-CpG probes, multi-hit probes, and probes located on chromosome X and Y were excluded. Beta-values, representing the methylation score for each CpG, were normalized using the Beta MIxture Quantile dilation (BMIQ) method [29]. To account for methylation differences between cell types, the cell-type composition was estimated using GLINT [30] and batch effect was corrected by ComBat method [31].

DNAm data (*N* = 724,619 probes remained) from whole blood samples were submitted to the online DNAmAge Calculator (https://dnamanage.genetics.ucla.edu/) and DNAm age was calculated. Four distinct EA estimates and estimates of EAA, including HorvathAgeAccel, HannumAgeAccel, PhenoAgeAccel, GrimAgeAccel, were calculated. HannumAgeAccel is an estimate derived from the Hannum methods based on 71 CpGs, and HorvathAgeAccel is derived from the Horvath method based on 353 CpGs that is independent of blood cell counts [20, 21]. GrimAge is an EA marker enriched for DNAm sites that are surrogate biomarkers for blood plasma proteins [32]. DunedinPACE, a DNAm biomarker of pace of aging, was calculated using a publicly available R package (https://github.com/danbelsky/DunedinPACE*).*

### Statistical analysis

Demographic, lifestyle, clinical, and sleep characteristics of study participants were expressed as means (SDs) or median (25th percentile, 75th percentile) for continuous variables and numbers (percentage) for categorical variables. The study participants were categorized into either good sleepers (the global PSQI score ≤ 5) or poor sleepers (the global PSQI score > 5). The differences between the groups were analyzed with independent t-test or Mann–Whitney U-test for normally distributed continuous variables or Kruskal–Wallis rank sum test for skewed distributed continuous variables. Chi-square test was used for categorical variables. Incomplete questionnaires missing the variables of interest were excluded from the analysis. Sex, age, smoking status, drinking status, and physical activity were considered potential confounding factors. A *p*-value threshold of 0.05 was set to determine statistical significance. Pearson’s correlation coefficients between each EAA (GrimAgeAccel, PhenoAgeAccel, HorvathAgeAccel, HannumAgeAccel, and DunedinPACE) and PSQI were calculated to evaluate prediction accuracy. To address the prediction model between PSQI and EAAs, linear regression models adjusted for covariates including chronological age, BMI, smoking, drinking, monthly income, and physical activity were applied. Subsequently, the associations between PSQI score and the incident of diseases (binary outcome) were evaluated using logistic regression. All statistical analyses were performed using R version 4.3.2 (The R Foundation, Vienna, Austria).

## Results

### Study participants and baseline characteristics

Of the total 701 study participants, who were asked to complete the questionnaire, 9 (1.3%) with at least one missing variable related to health conditions were excluded. Among the total 692 participants included for final analysis, 63.7% were categorized as good sleepers (*n* = 441) and 36.3% as poor sleepers (*n* = 251). Table 1 presents the baseline demographic, lifestyle, and clinical characteristics of the study participants according to their sleep quality. PSQI scores ranged between 0 and 19 with a median score of 4 (interquartile range [IQR], 3–6). The median chronological age of good sleepers (53.0 years, IQR 50.0–59.0) was not significantly different from that of poor sleepers (56.9, IQR 50–61.5). Compared to good sleepers, poor sleeper had higher proportion of females (48.2% vs. 34.5%, *p* < 0.01) and lower monthly income ($\le$ 200 million KRW) (29.9% vs. 22.7%, *p* < 0.05). Lifestyle behaviors (including physical activity, smoking status, and drinking status) and clinical conditions (including glycosylated hemoglobin A1c (HbA1C), HDL, TG, total cholesterol, FAG and BP) did not show significant differences between two groups.

**Table 1:** Baseline demographic, lifestyle, and clinical characteristics of the study participants according to the sleep quality

Characteristics	Total (*N* = 692)	Good sleepers (*N* = 441)	Poor sleepers (*N* = 251)	*p*-value
PSQI	4.0 (3.0–6.0)	3.0 (2.0–4.0)	7.0 (6.0–9.0)	< 0.01
Age, years	54.0 (50–60)	53.0 (50.0–59.0)	55.0(51.0–61.5)	0.08
Sex (%)				
Male	420 (60.6)	289 (65.5)	130 (51.8)	< 0.01
Female	273 (39.4)	152 (34.5)	121 (48.2)	
Monthly income (%)				
≤ 200 million KRW	175 (25.3)	100 (22.7)	75 (29.9)	< 0.05
> 200 million KRW	518 (74.7)	341 (77.3)	176 (70.1)	
BMI (kg/m^2^)	24.4 (22.6–26.2)	24.4 (22.7–26.4)	24.2 (22.6–26.0)	0.59
PA (MET-hour/day)	41.8(5.7)	42.2 (5.7)	41.0 (5.7)	0.43
Smoking status (%)				
Non-smoker	345 (49.8)	234 (47.7)	111 (55.0)	0.09
Former smoker	223 (32.3)	165 (33.6)	59 (29.2)	
Current smoker	124 (17.9)	92 (18.7)	32 (15.8)	
Drinking status (%)				
Non-drinker	345 (43.4)	209 (42.6)	92 (45.5)	0.45
Former drinker	224 (3.3)	16 (3.2)	7 (3.5)	
Current drinker	124 (53.3)	266 (54.2)	103 (51.0)	
HbA1C (%)	5.5 (5.3–6.2)	5.5 (5.3–6.1)	5.5 (5.3–6.4)	0.32
HDL (mg/dL)	42.0 (36.0–50.0)	42.0 (36.0–50.0)	42.0 (35.0–50.0)	0.89
Triglyceride (mg/dL)	118.0(85.0–171.2)	115.0 (84.0–167.0)	123.0(86.5–174.5)	0.72
Total cholesterol (mg/dL)	195.5(171.0–219.2)	195.0 (171.0–218.0))	198.0(172.0–222.0)	0.18
Fasting glucose (mg/dL)	94.0(88.0–107.2)	94.0 (88.0–107.0)	93.0 (87.0–110.0)	0.63
Blood pressure (mmHg)				
Systolic BP	113.0(105.0–123.0)	113 (106.0–124.0)	114.0(103.0–122.0)	0.18
Diastolic BP	76.0 (69.0–81.0)	76 (70.0–82.0)	76 (69.0–80.0)	0.10
Insulin (µIU/mL)	8.2 (6.2–10.7)	8.1(6.1–10.6)	8.5 (6.4–10.9)	0.25
hs-CRP (mg/L)	0.7 (0.3–1.2)	0.7 (0.4–1.2)	0.6 (0.3–1.3)	0.61
WBC (Thous/µL)	5.4 (4.5–6.5)	5.4 (4.6–6.5)	5.4 (4.5–6.5)	0.79
Chronic diseases				
Hypertension^a^ (%)	34 (4.9)	23 (5.2)	11 (4.4)	0.76
Diabetes^a^ (%)	21 (3.0)	9 (2.0)	12 (4.8)	0.07
Hyperlipidemia^a^ (%)	22 (3.2)	11(2.5)	11 (4.4)	0.26
Metabolic syndrome (%)	226 (32.7)	139 (31.5)	87 (34.7)	0.45

*PSQI* Pittsburgh Sleep Quality Index; *PA* physical activity; *MET* metabolic equivalent test; *BMI* body mass index; *KRW* Korean Won; *HbA1C* glycosylated hemoglobin A1c; *HDL* high-density lipoprotein; *hs-CRP* high sensitive-C reactive protein; *WBC* white blood cell count. Data are presented as median (25th percentile, 75th percentile) or mean (SD) for continuous variables and number (%) for categorical variables. ^a^diagnosed by medical doctor. Independent *t* test for normally distributed data or Willcoxon rank sum test for non-normally distributed data was used for continuous variables, and Chi-square test was used for categorical variables. Poor sleepers are PSQI score > 5

### Correlations between methylation age and sleep quality

**Fig. 1:**
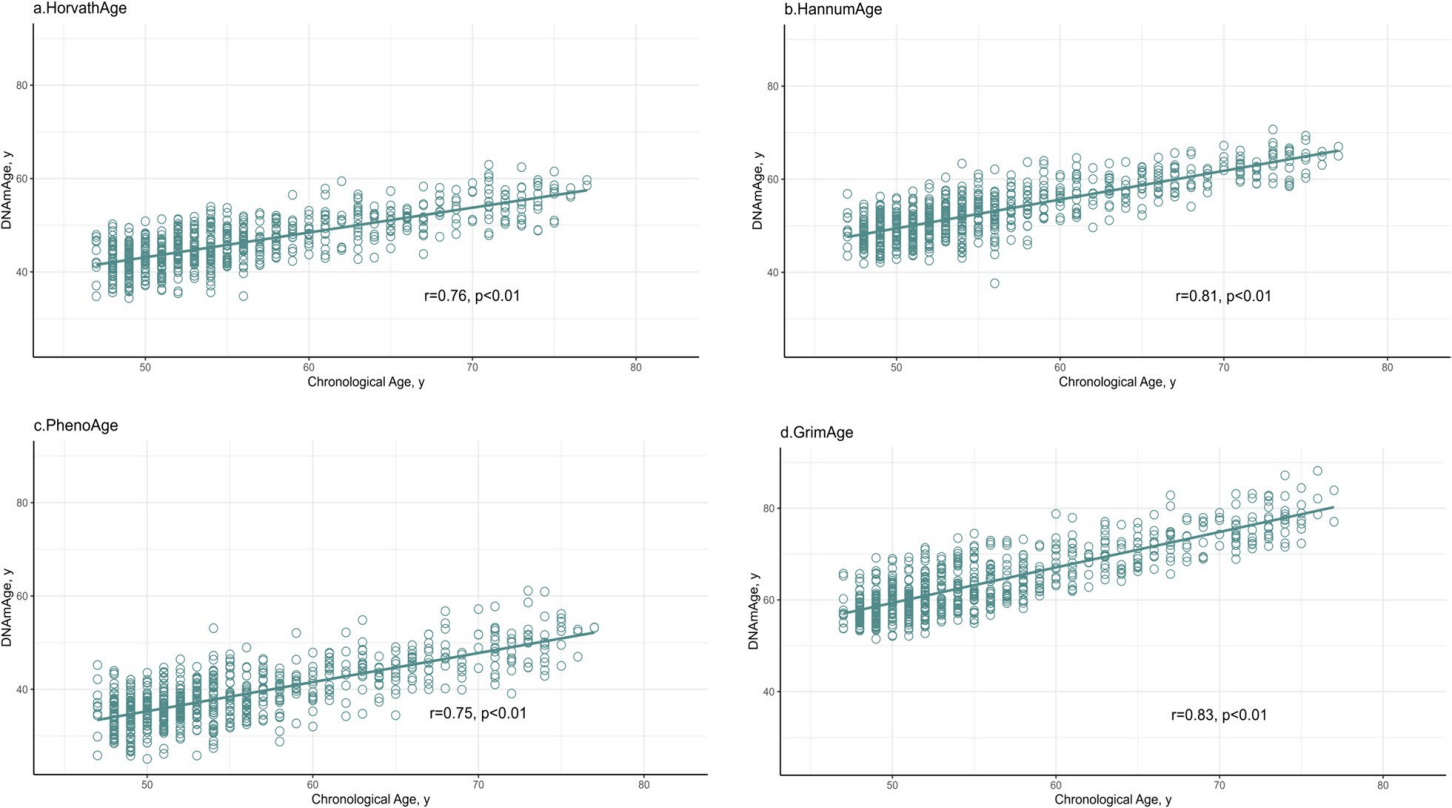
Correlation of chronological age and epigenetic ages among Korean adults. Chronological age correlates with various DNAmAge (HorvathAge, HannumAge, PhenoAge, and GrimAge). The figure shows scatter plots of chronological age (X-axis) against epigenetic age (Y-axis) of **a** HorvathAge, **b** HannumAge, **c** PhenoAge, and **d** GrimAge in whole blood

Significant correlations were observed between chronological age and each EAs: HorvathAge ($\gamma$= 0.76, *p* < 0.01), HannumAge ($\gamma$=0.81, *p* < 0.01), PhenoAge ($\gamma$=0.75, *p* < 0.01), and GrimAge ($\gamma$=0.83, *p* < 0.01). These results suggest a valid high accuracy of the epigenetic estimator used in this study (Fig. 1). There were no statistical differences in EA levels between good and poor sleepers (Table 2). The mean (SD) values of HorvathAge, HannumAge, PhenoAge and GrimAge were 46.43 (5.33), 53.28 (5.81), 39.14 (6.33), and 64.14 (7.07), respectively. Unexpectedly, the mean level of GrimAgeAccel was significantly higher in good sleepers compared to poor sleepers (0.65 vs. 0.03, *p* = 0.02).

**Table 2:** Methylation age and its acceleration between good and poor sleepers

EA or EAA	Total (*N* = 692)	Good sleepers (*N* = 441)	Poor sleepers (*N* = 251)	*p*-value
HorvathAge	46.43 (5.33)	46.30 (5.28)	46.60 (5.41)	0.25
HannumAge	53.28 (5.81)	53.2 (5.75)	53.51 (5.92)	0.12
PhenoAge	39.14 (6.33)	39.05 (6.29)	39.30 (6.41)	0.42
GrimAge	64.14 (7.07)	64.08 (6.96)	64.26 (7.26)	0.23
HorvathAgeAccel	0.80 (3.37)	0.82 (3.39)	0.76 (3.33)	0.93
HannumAgeAccel	0.82 (3.01)	0.79 (2.97)	0.88 (3.09)	0.31
PhenoAgeAccel	1.10 (4.23)	1.23 (4.23)	0.88 (4.22)	0.23
GrimAgeAccel	0.42 (3.94)	0.65 (3.88)	0.03 (4.03)	0.02
DunedinPACE	1.02 (0.08)	1.02 (0.08)	1.02 (0.09)	0.41

*AgeAccel* epigenetic age acceleration; *EA* epigenetic age; *EAA* epigenetic age accelerator. Data were shown as mean (standard deviation). *p-value* was calculated using *t* test or Mann–Whitney U-test

**Fig. 2:**
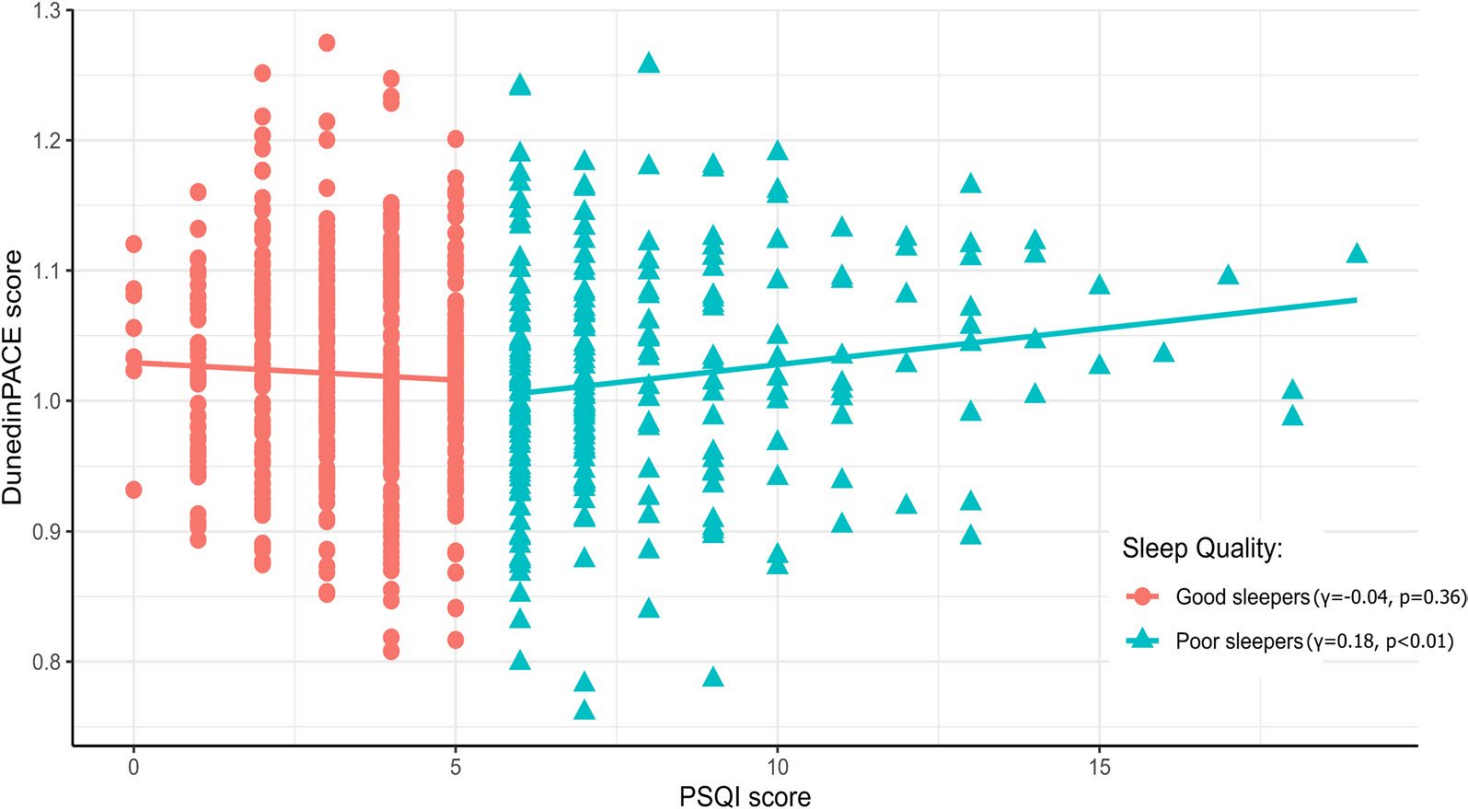
Correlation of DunedinPACE and PSQI score between good and poor sleepers. DunedinPACE was positively correlated with PSQI scores in poor sleepers ($\gamma$ =0.18, *p* < 0.01 for poor sleepers; $\gamma$ = − 0.04, *p* = 0.36 for good sleepers). PSQI, Pittsburgh Sleep Quality Index

Correlation between EAAs and PSQI scores was investigated, stratified by sleep quality (good vs. poor). DunedinPACE was positively correlated with PSQI scores in poor sleepers (Fig. 2, $\gamma$=0.18, *p* < 0.01 for poor sleepers; $\gamma$=$-$ 0.04, *p* = 0.36 for good sleepers). No association was observed between other DNA methylation accelerations and PSQI scores in either group (Supplementary Fig. 1).

### Linear regression models between methylation age accelerators and sleep quality

Given the positive correlation observed between DunedinPACE and PSQI scores in poor sleepers, unique predictive power of sleep quality on different EAAs was further investigated using different regression models. Two different prediction models were established through linear regression of EAAs against PSQI scores: Model 1 was adjusted for sex as a covariate. Model 2 was adjusted for chronological age, smoking status, drinking status and BMI, in addition to sex. In all models, GrimAgeAccel and DunedinPACE showed a statistically significant association with PSQI scores only in poor sleepers (Table 3).

**Table 3:** Linear regression analysis for PSIQ score and methylation age acceleration between good and poor sleepers

Age accelerators	Good sleepers (*N* = 441)			Poor sleepers (*N* = 251)		
	*ꞵ*	SE	*p-value*	*ꞵ*	SE	*p-value*
Model 1						
HorvathAgeAccel	− 0.098	0.122	0.422	0.085	0.083	0.307
HannumAgeAccl	− 0.126	0.107	0.238	0.137	0.077	0.079
GrimAgeAccel	0.111	0.110	0.315	0.179	0.076	1.94 $\times {10}^{-2}$
PhenoAgeAccel	0.016	0.151	0.918	0.114	0.106	0.285
DunedinPACE	− 0.002	0.003	0.533	0.006	0.002	5.54 $\times {10}^{-3}$
Model 2						
HorvathAgeAccel	0.082	0.107	0.446	0.071	0.085	0.402
HannumAgeAccl	− 0.118	0.108	0.273	0.100	0.076	0.188
GrimAgeAccel	0.078	0.099	0.430	0.167	0.066	1.16 $\times {10}^{-2}$
PhenoAgeAccel	0.022	0.154	0.887	0.082	0.107	0.446
DunedinPACE	− 0.001	0.003	0.960	0.004	0.002	3.63 $\times {10}^{-2}$

Model 1 presents the results from linear regression model analysis of PSQI on methylation age accelerator (dependent variables) adjusted for sex. We additionally adjusted for chronological age, smoking status, drinking status, and BMI (Model2)

### Associations between PSQI scores and MetS risk in poor sleepers

Table 4 presents the associations between PSQI scores and the incidence of chronic diseases, including type2 diabetes, hypertension, hyperlipidemia and MetS in poor sleepers. The results showed that there were no relationships between PSQI scores and diabetes, hypertension, and hyperlipidemia in poor sleepers in both Model 1 (adjusted for chronological age and sex) and Model 2 (adjusted for chronological age, sex, smoking status, and DunedinPACE). However, in both models, significant associations were observed between PSQI and risk of MetS in poor sleepers. For every one-point increase in PSQI score, there was a 16% increase in the risk of MetS in poor sleepers (Model 2 OR = 1.16, 95% CI = 1.04–1.30, *p* < 0.01).

**Table 4:** Risk of type 2 diabetes, hypertension, hyperlipidemia, and metabolic syndrome by PSQI in poor sleepers

Chronic diseases	Poor sleepers (*N* = 251)		
	OR	95% CI	*p-value*
Model 1			
Type2 diabetes	1.02	0.76, 1.27	0.89
Hypertension	1.10	0.87, 1.33	0.36
Hyperlipidemia	1.08	0.85, 1.31	0.49
Metabolic syndrome	1.18	1.06, 1.32	< 0.01
Model 2			
Type2 diabetes	1.01	0.75, 1.26	0.96
Hypertension	1.07	0.84, 1.31	0.50
Hyperlipidemia	1.06	0.82, 1.30	0.62
Metabolic syndrome	1.16	1.04, 1.30	< 0.01

*OR* odds ratio; *CI* confidence interval. Model 1 is adjusted for chronological age and sex. Model 2 includes chronological age, sex, smoking status, and DunedinPACE as covariates

Additionally, the association between PSQI scores and each MetS component in poor sleepers was investigated. A positive correlation was observed between PSQI score and FAG ($\gamma$= 0.19, *p* < 0.01).

## Discussion and conclusions

With the continuous increase in life expectancy, there is a growing interest in healthy aging, which depends on the interplay of the physiological, psychological, social, and environmental conditions. To date, multiple studies have suggested that biological age is a strong risk factor for various aging-related diseases such as chronic, metabolic, and neurodegenerative diseases. Biological aging is a complex phenomenon involving a multitude of biological processes from various organs and systems. Over the past decades, biological age has been estimated using a variety of biomarkers from our body at the cellular level [33]. Accumulating evidence indicates that epigenetic regulations such as DNA methylation, chromatin remodeling, and RNA modification play important roles in the aging process. DNA methylation ages with different algorithms were associated with inflammation, age-related health outcomes and mortality. Currently, the most comprehensive and accurate biological age test can be made through investigating epigenetic changes, such as epigenetic clocks in blood samples [33].

Sleep disturbance may be a candidate driver of the biological aging process. Given the increasing body of evidence linking sleep disruption to a magnitude of health issues, it is important to understand sleep as a vital physiological process. Sleep involves in many types of tissues and body systems, affecting circadian rhythms, hormone regulations, the immune system, and metabolic processes [34, 35]. Insufficient sleep disrupts critical neural processes and can lead to brain disorders [36]. Notably, insufficient sleep has been linked to the altering of physiological homeostasis and the development of several chronic diseases and conditions, including diabetes, cardiovascular disease, obesity, and mental health disorders [6, 37]. Recently, Kang et al. reported a significant association between poor sleep quality (PSQI > 5), shorter sleep durations (< 6 h), and MetS in the older age group (≥ 40 years) in a Korean population [38].

A few studies have investigated the association between sleep quality and accelerating biological aging. Decreased sufficient sleep duration is associated with accelerated epigenetic clocks in older females [39], during the postpartum period in women [14], and among freshmen in university [16]. Sleep physiology undergoes changes with the age, and many sleep disorders become more prevalent in the elderly. While differences in sleep patterns exist between younger and older adults, the aging process varies depending on sleep quantity and quality [40]. Decreased sleep latency, or taking a shorter time to fall asleep, is associated with increased longevity among centenarians [41]. Recently, Gau et al*.* reported that poor sleep quality, as determined by their own algorithm, was a causal factor in the acceleration of biological aging, indicated by KDM-biological age and PhenoAge [42]. While numerous factors related to aging have been investigated, the aspect of sleep quality has seemingly been overlooked until now. Therefore, we selected four aging acceleration markers and DunedinPACE as markers for biological age, which may be associated with sleep quality. In our study, we identified that the extent of poor sleep quality across seven major domains may be involved in accelerating biological ages of GrimAge and DundinPACE. In line with this finding, we observed that people with worsening sleep index in poor sleepers were associated with an increased risk of MetS [43]. Another study found that 62% of individuals with glucose levels in the pre-diabetes range were likely to have poor sleep, compared to 46% of individuals with normal glucose levels [44]. Disturbed sleep patterns have a significant impact on how the body processes glucose after meals, underscoring impaired glucose metabolism [45]. Given this observation, biological age estimation may provide a good candidate marker for investigating the biological mechanisms underlying the interplay between sleep- and aging-related health outcomes. One of the strengths of our study is that we used nationally representative samples and a relatively large sample size with validated assessment of sleep quality (Cronbach’s alpha = 0.7). However, there are several limitations in this study. Firstly, we did not assess the direction of causality between EAA and sleep quality using genetic determinants. Identifying a causal role of sleep conditions would be beneficial in elucidating biological mechanisms and providing potential preventive and treatment strategies. In addition, the epigenetic aging markers used in our study are not consistent with previous findings [42], which suggested that PhenoAgeAccel could have a causal effect on sleep duration. We hypothesize that differences in study ethnic groups and the assessment of sleep quality may have caused the inconsistent results. Further validation of our findings in more diverse populations will enhance the generalizability of our study. Even though the PSQI is one of the most widely used tools for assessing subjective sleep quality, PSQI does not provide objective physiological measurements for sleep quality.

In this study, worsening sleep quality, particularly among poor sleepers, is associated with accelerated epigenetic aging as indicated by GrimAgeAccel and DundinePACE, which in turn increases the risk of MetS including FAG. Our findings can potentially serve as a promising strategy for preventing age-related diseases in the future.

### Supplementary Information

Additional file 1.

## Data Availability

The Korean Genome and Epidemiology Study (KoGES) Consortium datasets in the current study are third-party data and are available under the approval of the data access committee of the National Biobank of Korea, who can be contacted at http://biobank.nih.go.kr (82-1661-9070).
