# Supplementary figures and images for: The association between sleep quality and accelerated epigenetic aging with metabolic syndrome in Korean adults
Source: Clin Epigenetics. 2024 Jul 16;16:92. doi: 10.1186/s13148-024-01706-x (PMC11253334; doi:10.1186/s13148-024-01706-x)

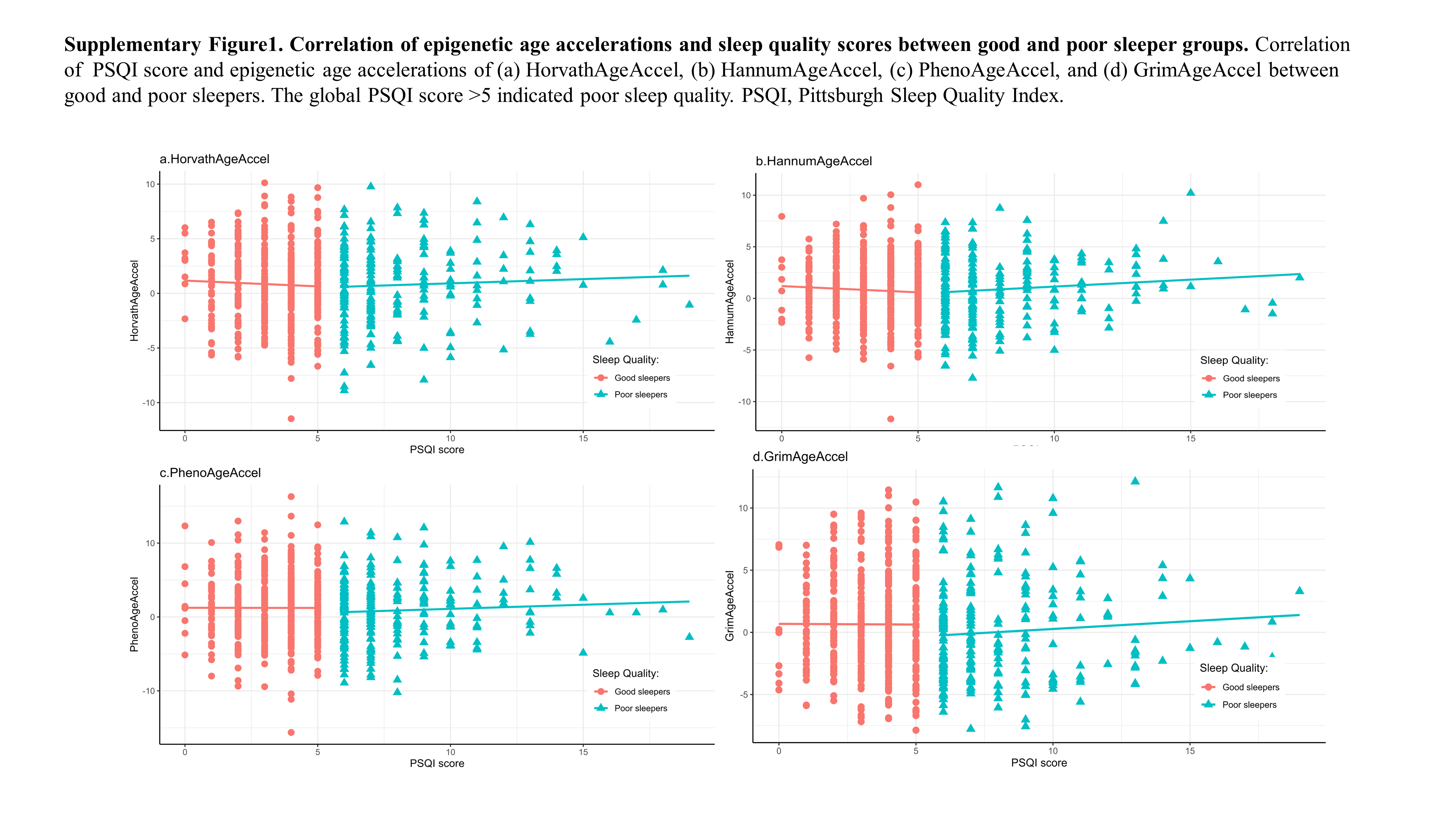

Supplement: Supplementary file 1 — Additional file 1. [file 13148_2024_1706_MOESM1_ESM.png]
